# A 3D Lattice Modelling Study of Drying Shrinkage Damage in Concrete Repair Systems

**DOI:** 10.3390/ma9070575

**Published:** 2016-07-14

**Authors:** Mladena Luković, Branko Šavija, Erik Schlangen, Guang Ye, Klaas van Breugel

**Affiliations:** Faculty of Civil Engineering and Geosciences, Delft 2628 CN, The Netherlands; b.savija@tudelft.nl (B.Š.); erik.schlangen@tudelft.nl (E.S.); g.ye@tudelft.nl (G.Y.); k.vanbreugel@tudelft.nl (K.v.B.)

**Keywords:** concrete repair, drying shrinkage, lattice model, strain hardening cementitious composites (SHCC)

## Abstract

Differential shrinkage between repair material and concrete substrate is considered to be the main cause of premature failure of repair systems. The magnitude of induced stresses depends on many factors, for example the degree of restraint, moisture gradients caused by curing and drying conditions, type of repair material, etc. Numerical simulations combined with experimental observations can be of great use when determining the influence of these parameters on the performance of repair systems. In this work, a lattice type model was used to simulate first the moisture transport inside a repair system and then the resulting damage as a function of time. 3D simulations were performed, and damage patterns were qualitatively verified with experimental results and cracking tendencies in different brittle and ductile materials. The influence of substrate surface preparation, bond strength between the two materials, and thickness of the repair material were investigated. Benefits of using a specially tailored fibre reinforced material, namely strain hardening cementitious composite (SHCC), for controlling the damage development due to drying shrinkage in concrete repairs was also examined.

## 1. Introduction

Concrete repair implies integration of the new (repair) material with the old concrete substrate in order to form a composite system capable of enduring exposure to mechanical loads and varying environmental conditions. What makes this difficult is the mismatch in age, properties, and performance of the two materials, often leading to premature failure of the multi-layered system [1,2]. Differential shrinkage, primarily drying shrinkage, is considered to be the major cause of failure [3,4].

After casting, the repair material is usually covered or coated in order to enable good curing and prevent plastic shrinkage cracking. After such curing, the repair system is uncovered and exposed to ambient temperature and relative humidity. Due to environmental drying, ongoing hydration, and moisture absorption by the concrete substrate, the repair material loses water and tends to shrink. Its deformation is, however, restrained by the concrete substrate. As a consequence, stresses build up in the repair system and cracking and/or debonding follows. Cracking or debonding of the overlay reduces the load-carrying capacity of the system and allows water, together with hazardous substances such as chloride, to penetrate into concrete and further speed up the deterioration process.

In order to make some practical design recommendations, a number of analytical models for bonded overlays subjected to differential shrinkage have been developed [4,5,6,7,8]. Also, a number of two-dimensional continuum models were used [3,9,10,11,12,13]. One way of coupling moisture transport and fracture simulations, while taking into account the influence of drying on cracking mechanism, is through discrete lattice type modelling [14,15]. The main disadvantage of all these numerical models is that restrained shrinkage deformation is studied mostly in 2D while the experiments are always 3D. In previous work with lattice approach [15], although a 3D mesh was used, the thickness of the simulated specimen was small in order to limit computational costs. Applied boundary conditions resulted in deformation of the material being restrained in one direction only, which does not completely agree with the behaviour in the experiments. Therefore, in order to further verify the applied modelling approach, cracking due to drying shrinkage in concrete repair systems needs to be simulated in 3D.

The aim of this paper is to understand parameters that influence shrinkage induced cracking by modelling moisture transport and fracture processes during drying in concrete repair systems. 3D mesoscale lattice models are used for simulating moisture distribution and resulting damage development in repair systems. Benefits of using lattice models for fracture simulations are that they can mimic physical structure and processes, so that realistic crack patterns can be achieved [16,17,18]. Such models have been used for modelling fracture of quasi-brittle (i.e., concrete, mortar or cement paste), but also ductile (i.e., fibre reinforced) materials [19,20]. In the presented models, the material structure is explicitly represented; in the lattice transport model it is captured through assigning different diffusivity (conductivity) properties [21,22,23], and in the lattice fracture model through assigning different mechanical properties [16] to lattice elements that represent a certain material phase. The influence of different parameters—for example substrate surface preparation (i.e., roughness), bond strength between the two materials, and thickness of the repair material on damage caused by drying shrinkage in concrete repair—were investigated.

The damage in the repair system can be reduced if the repair material is reinforced with fibres, as in case of strain-hardening cementitious composite—SHCC [24]. Fibres bridge crack faces and prevent large crack openings. In SHCC these cracks successively form and are limited to 100 micrometres. With successive microcracking in SHCC, load that can be transferred increases, implying that strain hardening behaviour, commonly observed in metals, is achieved. For applications as a repair material, multiple cracks of SHCC are beneficial for relief of stresses induced by deformational incompatibility between the repair material and the substrate [24,25]. Furthermore, dense microcracking and local debonding lead to high ductility of SHCC in repair systems, which is beneficial in suppressing crack localization due to flexural loading [26,27]. In this paper, implications of using SHCC as a repair material on damage caused by restrained drying shrinkage are addressed. Simulated 3D damage patterns in repair systems with different types of repair materials, their thickness, and bond strength are compared with experimental observations and cracking tendency in various brittle and ductile materials.

## 2. Modelling Approach

Lattice models have been widely used to simulate fracture, moisture transport, and chloride diffusion in cement-based materials [14,16,21,23]. In the transport lattice approach, concrete is treated as an assembly of one-dimensional “pipes”, through which the flow takes place. In the mechanical lattice approach, concrete is discretized as a set of truss or beam elements that transfer forces. The output from the moisture transport model is used as an input for simulating fracture. This is a staggered flow-stress analysis: moisture transport induces strain which might result in cracking, but there is no influence of the (micro)cracking on the moisture transport. This is a reasonable assumption as drying shrinkage microcracks seem not to have a significant effect on drying rate of mortars and concrete [28]. With wetting and drying cycles, however, cracks may have a significant influence, and transport and fracture simulations would probably need to be fully coupled.

### 2.1. Spatial Discretization

**Figure 1:**
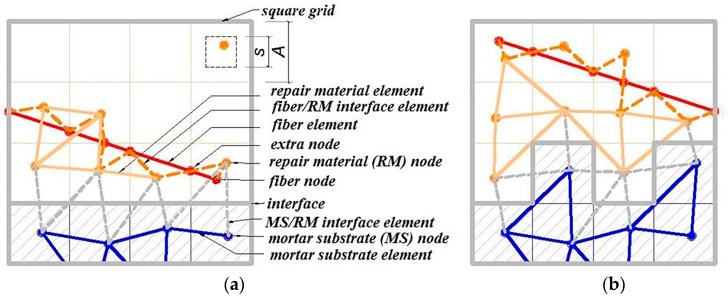
Two-dimensional overlay procedure for generation of the lattice model in the interface zone between the fibre reinforced repair material and (**a**) a smooth; and (**b**) a rough surface substrate.

**Figure 2:**
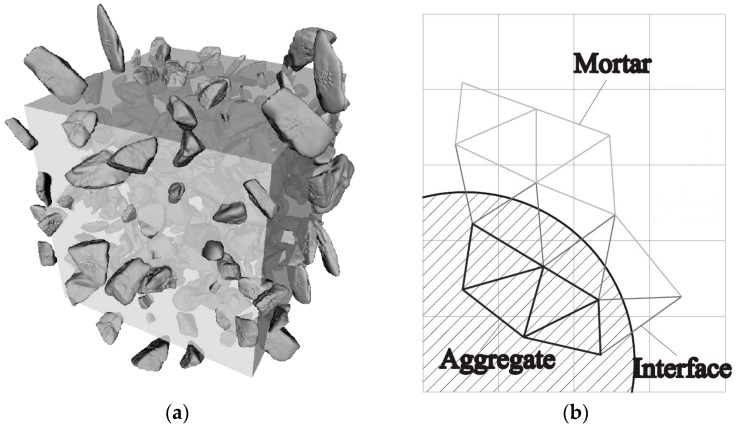
Including material mesostructure of the concrete substrate (**a**) aggregates in the concrete substrate generated by Anm model [32]; (**b**) two-dimensional overlay procedure for generation of the lattice model in the interface zone between aggregate and substrate mortar (ITZ).

**Figure 3:**
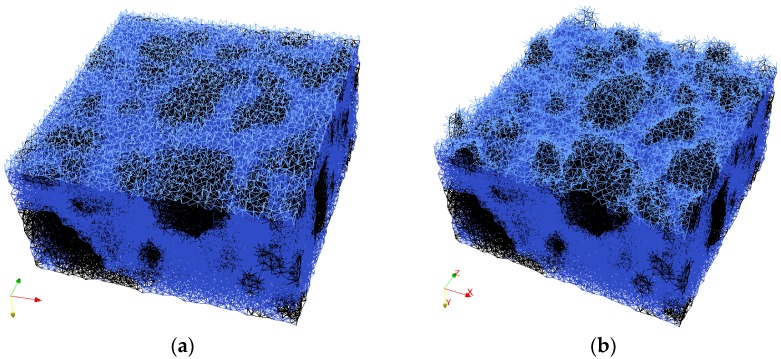
Generated lattice model for (**a**) a smooth; and (**b**) a “sandblasted” surface substrate.

**Figure 4:**
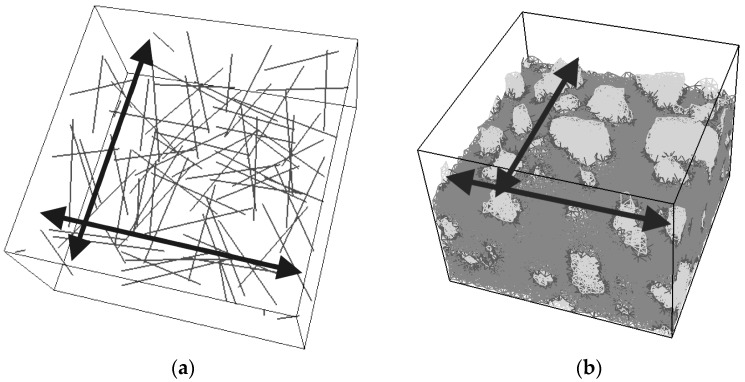
Periodic boundary conditions in horizontal directions for (**a**) fibres (**grey** colour) in the SHCC repair material (**b**) aggregates (**light grey** colour) in the concrete substrate.

The approach proposed here uses the same lattice network for both the moisture and the fracture simulations. For the spatial discretization of the specimen in three dimensions, the basis is the prismatic domain [21]. Discretization of the domain was performed according to the following procedure:

- A cubical grid was chosen (square for 2D lattice) and the domain was divided into a number of cubical cells (Figure 1a,b and Figure 2b). In all simulations presented herein, linear dimension of the cubical cell *A* (marked in Figure 1a) was 1 mm.
- In each cell of size *A*, a sub cell with linear dimension of *s* was defined (marked in Figure 1a). The lattice node was randomly positioned inside this sub cell (Figure 1a). The ratio *s*/*A* is defined as randomness of a lattice. When randomness is 0, the node is located in the centre of cell and regular lattice mesh is generated. If the randomness is higher than 0, beams have different lengths and material disorder is built through the geometry of lattice mesh. With randomness of 1, therefore, materials have the maximum degree of disorder. The choice of randomness affects the simulated fracture of materials to a certain extent, as different orientation of meshes can affect the crack shape [29]. In order to include benefits of random mesh, the randomness here was set to be 0.5.
- Voronoi tessellation of the prismatic domain with respect to the specified set of nodes was performed. Nodes with adjacent Voronoi cells were connected by lattice elements (Figure 1a,b and Figure 2b). Since Voronoi diagrams are dual with Delaunay tessellation, this approach is equivalent to performing a Delaunay tessellation of the set of nodes [21].
- In order to take material heterogeneity into account, either a computer-generated material structure, or a material structure obtained by micro-CT scanning [17,30] can be used. Here, the concrete mesostructure was simulated using the Anm material model originally developed by Garboczi [31] and implemented in a 3D packing algorithm by Qian et al. [32]. It is based on placing multiple irregular shape particles separated into several sieve ranges into a predefined empty container (Figure 2a). Aggregates smaller than 4 mm in the substrate are not explicitly modelled and, together with cement paste, are considered to be part of the mortar phase. Similarly, in the repair material, the largest sand (filler) particles are around 200 μm and, as such, are also not explicitly simulated.
- Material overlay procedure (schematically shown in Figure 1a,b and Figure 2b) was employed: the beams that belong to each phase were identified by overlapping the material mesostructure (i.e., substrate mortar, repair material mortar, and aggregates) on top of the lattice. Interface elements between the repair material and the substrate for smooth and rough substrate surface were generated between substrate nodes and repair material nodes (Interface MS/RM, Figure 1a,b, respectively). Aggregate-mortar interface (ITZ) elements were generated between mortar nodes and aggregates (Figure 2b). By applying this overlay procedure, the geometrical roughness of the substrate was also explicitly modelled (Figure 3a,b).
- Different transport and mechanical properties were assigned to different phases (Table 1 and Table 2, respectively). Interfaces, as used in the present model, are always considered as one row of beam elements. As such, they do not exactly coincide with the size of real interfaces. In reality, interface thickness is in a range of tens of micrometres, while interface elements in the present model also take up a piece of aggregate (or repair material) and a piece of mortar. Therefore, the actual size of the interface in the model depends on the characteristic element size and, in presented simulations, is around 1 mm.
- For fracture simulations, fibre elements were added in the repair material with a design volume content (0.5%), fibre length (8 mm) and diameter (0.04 mm). The location of the first node of each fibre was chosen randomly in the specified volume and a random direction was defined which determined the position of the second node (Figure 4a). If the second node was outside the mesh boundary, then the fibre was automatically cut off.
- Extra nodes inside the fibres were generated at each location where the fibre crosses the grid (Figure 1a,b).
- Fibre/matrix interface elements were generated between fibre nodes and the matrix nodes in the neighbouring cell. Also, the end nodes of the fibres were connected with an interface element to the matrix node in the cell where the fibre end was located (Figure 1a,b).
- Note that in order to reduce the computational time, 0.5% instead of 2% of fibres, as commonly used in SHCC, was simulated. In order to obtain SHCC fracture behaviour in simulations with a lower percentage of implemented fibres, fibre/matrix interface strength and fibre tensile strength from Table 2 are higher compared to experimentally measured values which can be found in [27].
- Samples were simulated with periodic boundary conditions. This means that one side of the specimen was connected to the other end. Therefore, aggregates and fibres were distributed in such a way that they continue through boundaries and periodically repeat. The example for the periodic boundary conditions of the fibres is given in Figure 4a and for aggregates in Figure 4b.
- Fibre elements and fibre/matrix interface elements were assumed not to take part in the moisture transport and therefore are not modelled in the transport simulations.
- In order to have a representative volume, the length of the repair system was chosen to be at least 5 times larger than the maximum aggregate size and fibre length in the repair material.

### 2.2. Lattice Moisture Transport Model

A random lattice (*s*/*A* = 0.5) was used first to model moisture transport caused by water exchange in a repair system. The model treats concrete as an assembly of one-dimensional, linear, “pipe” elements, through which moisture transport takes place. A governing partial differential equation for moisture transport in 1D is:

(1) $$\frac{\partial H}{\partial t}=\frac{\partial }{\partial x}\left(D\left(H\right)\frac{\partial H}{\partial x}\right)$$

where *H* is the relative humidity and *D*(*H*) is a humidity dependent diffusion coefficient which can be given as:

(2) $$D\left(H\right)=\beta e^{\gamma^{H}}$$

Here *β* and *γ* are parameters that can be determined by calibration with experimental results [33]. In the model presented herein, although moisture transport is in reality a multi-scale problem, no distinction was made between the different flow mechanisms that take place at the micro-scale [34].

**Figure 5:**
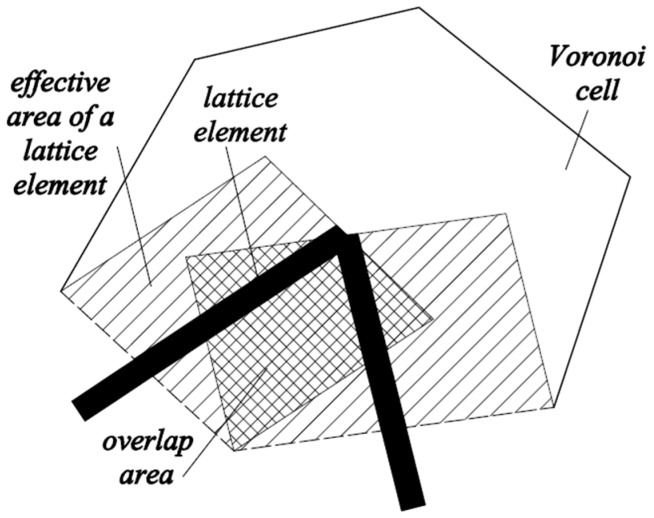
Definition of overlap area for determination of parameter *ω* (adapted from [34]).

If Equation (1) is discretized using the standard Galerkin method [35], the following set of linear equations arises (in matrix form):

(3) $$M\dot{H}+KH=f$$

where *M* = mass matrix; *K* = diffusion matrix and *f* = force vector. Vector *H* is the vector of unknown quantities (in this case relative humidity) and dot over *H* indicates the time derivative. Element matrices, *M_e_* and *K_e_*, have the following forms:

(4) $$M_{e}=\frac{A_{ij}l_{ij}}{6\omega }\left[\begin{array}{cc} 2 & 1\\ 1 & 2 \end{array}\right]\;\;\;\;\;\;\;K_{e}=\frac{D\left(H\right)A_{ij}}{l_{ij}}\left[\begin{array}{cc} 1 & -1\\ -1 & 1 \end{array}\right]$$

where *l_ij_* is the length of the element between nodes *i* and *j*, *A_ij_* is its cross sectional area, and *D*(*H*) its diffusion coefficient. Cross sectional areas of individual elements were assigned using the so-called Voronoi scaling method [36]. All matrices were equivalent to those of regular one-dimensional linear elements [35], except the correction parameter *ω* in the mass matrix (Equation (4)). This parameter was used to convert the volume of all lattice elements to the volume of the specimen, due to overlap of volume of adjacent lattice elements (Figure 5). Therefore, *ω* corresponds to the ratio between the total area of Voronoi facets through which moisture transport takes place and the volume represented by lattice mesh, and can be determined as [37]:

(5) $$\omega =\frac{\sum_{i=1}^{m}A_{i}l_{i}}{V}$$

where *m* is the total number of elements, *A_i_* and *l_i_* cross sectional area and length of each lattice element, *k* element number, and *V* the total volume of the specimen. In this way, the assemblage of lattice elements in the mesh provided 3D moisture simulations by using 1D moisture flow in lattice elements.

When flux, *q_s_*, occurs between the material boundary and the atmosphere, it is necessary to account for convective boundary conditions:

(6) $$q_{s}=C_{f}\left(H_{s}-H_{a}\right)$$

Here, *C_f_* is the film coefficient, *q_s_* is the moisture flux across the boundary, *H_s_* and *H_a_* are the relative humidities at the material surface and surrounding atmosphere, respectively. In the lattice model, the evaporation rate was implemented through force vector in the element *f_e_*:

(7) $$f_{e}=\frac{q_{s}A_{ij}}{\vartheta }\left[\begin{array}{c} 1\\ 0 \end{array}\right]=\frac{C_{f}A_{ij}}{\vartheta }\left[\begin{array}{c} H_{s,i}-H_{a}\\ 0 \end{array}\right]$$

where *H_s,i_* is the relative humidity of the surface node (node at the surface exposed to drying) and *ϑ* is the correction factor which is determined as:

(8) $$\vartheta =\frac{\sum_{i=1}^{n}A_{s}}{A}$$

Here, *n* is the total number of elements corresponding to the surface nodes, *A_s_* is the area of Voronoi facets corresponding to these elements and *A* is the total area of the surface exposed to drying. The concept is similar as for determination of the correction factor *ω* (see Equation (5)).

Using the Crank-Nicholson procedure [35], the system of linear equations was then discretized in time, and the following equation resulted:

(9) $$\left(M+0.5∆tK^{n-1}\right)H^{n}=\left(M-0.5∆tK^{n-1}\right)H^{n-1}+∆tf$$

This equation was then solved for each discrete time step (∆*t*) and moisture contents were obtained. Since parameter *D* and, therefore, *K* is dependent on relative humidity *H*, an iterative procedure was avoided by calculating the relative humidity in each step (*n*) based on values of hydraulic diffusivity determined from the previous step (*n* − 1). Although this implies a certain amount of error, it significantly shortens the simulation time and the error is small as long as the time step ∆*t* is kept small. A benefit of using a lattice type moisture model is that further fracture simulations are coupled with moisture simulations whilst the same material mesostructure and mesh are used.

#### Transport Properties of Lattice Elements

For the analysis of the time-dependent drying shrinkage, a repair system was simulated. A similar problem was studied numerically by Wittmann and Martinola [12], Sadouki and van Mier [11], and Bolander and Berton [14]. Therefore, the same input parameters were used: the system was exposed to environment with 50% relative humidity (*H_a_* = 0.5) and film coefficient of the surface was *0.7* mm/day. The terms in the diffusivity Equation (2) and Table 1 were set to obtain humidity profiles similar to those obtained by Martinola and Wittmann [12]. The concrete substrate was pre-saturated, which means that the top layer had the same initial relative humidity as the repair material (*H* = 1).

Concrete substrate mesostructure was included. The volume percentage of the crushed stone in the simulated concrete substrate was 30%. This should approximately correspond to the amount of coarse aggregates in the concrete, while the sand and cement paste were considered to form a mortar phase. Sixty-seven percent (67%) of coarse aggregates were in the sieve range of (8,16) mm and 33% in the sieve range of (4,8) mm. In the present model, aggregates were assumed to be impermeable (as similarly done by Wang and Ueda when modelling capillary absorption of concrete in [23]) and were ascribed very low diffusivity properties (Table 1). It has to be highlighted however, that for different type of aggregates, and especially for lightweight aggregates, this assumption is not valid. The surface nodes of aggregates, which are in contact with surrounding matrix, have the same initial relative humidity as the surrounding matrix. As a zone with higher porosity, ITZ has higher diffusivity properties than the bulk matrix. In current simulations, 3 times higher diffusivity compared to the bulk matrix diffusivity is ascribed to ITZ. This is in line with ratio used by Šavija et al. [21] for chloride diffusivity. In their study, effective diffusion rate for chloride penetration in various concrete mixtures was calculated, and 2.5–7 times higher diffusion coefficient for ITZ elements compared to bulk matrix was obtained. Since both chloride diffusivity and water movement are directly related to the porosity, the same ratio was assumed to be valid in this study. In this way, effective diffusivity for the ITZ elements (which depends on the chosen mesh size) was fitted from experiments. It might, however, be argued whether the same ratio for moisture transport and chloride penetration can be used in these simulations. Wang and Ueda [23], for example, used 10 times higher diffusivity of ITZ elements when modelling moisture absorption of the concrete. Note that in their simulations, however, the thickness of ITZ was around 50 micrometres, while interface simulated herein was much thicker (around 1 mm). Therefore, the assumed ratio of 3 is probably a reasonable starting point but should be calibrated with experimental results for the specific mixture.

To check the accuracy of the model, non-linear solution procedure, and implementation of convective boundary conditions, simulation results from the lattice moisture model were previously compared with those from a commercial finite element model FEMMASSE MLS [38] and published in [15]. The lattice model showed performance comparable to the commercial FE model.

Note that, for different water-to-cement ratios and aggregate content of the materials, diffusion parameters (as given in Table 1) would be different. In order to obtain moisture diffusivity experimentally, concrete substrate and repair mortar should be exposed to different environmental conditions, and an inverse numerical technique should be applied on processing the experimental data [12]. Therefore, for different repair materials and substrates, moisture transport properties need to be measured and used as input for the simulation.

### 2.3. Lattice Fracture Model

In lattice fracture models, the material is discretized as a network of beam elements connected at the ends. All individual elements have linear elastic behaviour. In each loading step, an element that exceeds the limit stress capacity is removed from the mesh. The analysis procedure is then repeated until a pre-determined failure criterion is reached.

For the fracture criterion, only axial forces are taken into account to determine the stress in the beams. An element in the lattice fracture model can fail either in tension or in compression, when the stress exceeds its strength.

#### Mechanical Properties of Lattice Elements

In fracture simulations, different mechanical properties were ascribed to elements of a certain phase (Table 2). Two different mixtures of repair material were simulated. First, repair material was simulated as a repair mortar, with homogenous local properties. In the other mixture, PVA (polyvinyl alcohol) fibres were added. These fibres enable crack bridging and a strain hardening behaviour of a material, without a significant change in tensile strength. Such a strain-hardening cementitious composite (SHCC) can be used as a repair material. SHCC was first tailored through a single fibre pull-out test and then tested in a direct tension test. More details about the procedure can be found in [27].

Mechanical properties of interface elements, which connected the repair material and mortar substrate used in the simulations, were assumed values. As the interface is usually the weakest zone in the system, lower properties were ascribed to elements that characterize this zone. To investigate the influence of interface strength on the fracture behaviour of the repair system, the tensile strength of the interface is varied from 1 to 3 MPa.

For simplicity, it was assumed that the concrete substrate strength is similar to the repair material strength. Although in real repair systems this is not the case, here it was done in order to avoid additional influence of this parameter on the response of the system. Properties of ITZ and mortar in the concrete substrate were, therefore, adjusted so that the tensile strength of the substrate was similar to the strength of the repair material. It has to be highlighted, however, that assumed substrate strength will affect the balance between debonding and cracking in repair material and/or substrate, as discussed in Chapter 7 in [39].

### 2.4. Coupling of the Lattice Moisture Model with the Lattice Fracture Model

For coupling moisture and fracture analysis, it can be assumed that the moisture distribution produces a shrinkage field according to equation [3]:

(10) $$\varepsilon_{sh}=\alpha_{sh}∆H$$

where *ε_sh_* is the unrestrained shrinkage strain, Δ*H* is the change in relative humidity and *α_sh_* hygral coefficient of shrinkage which can be measured from drying tests at different relative humidities. Hygral coefficients were taken from experimental measurements of Martinola and Wittmann [12]: for repair material *α_sh,RM_* = 0.0048; for interface between repair material and substrate *α_sh,INT_* = 0.0028; and for substrate *α_sh,SUB_* = 0.0013. Note that, for different mixtures of repair material and concrete substrate, hygral coefficients will differ and should be measured experimentally.

**Figure 6:**
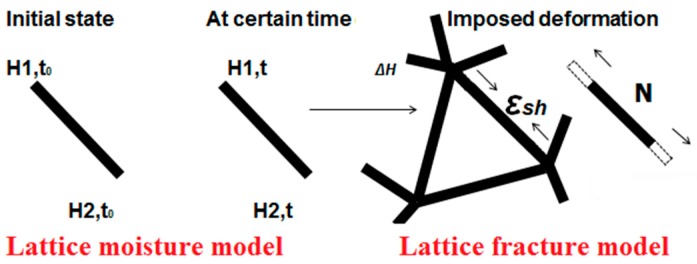
Coupling lattice moisture and lattice fracture model in time (*H*-relative humidity).

If a material is completely free to deform, shrinkage deformation due to moisture change results only in volume change of the material and no stress occurs. However, internal restraints in material (i.e., aggregates, stiff inclusions) and external restraints (i.e., bond with the substrate) result in eigenstresses and local stress concentrations that might exceed the material strength. Once the strength is exceeded, cracking in the material occurs. The lattice mesh mimicked this behaviour. Due to the relative humidity change (Δ*H*), every element tended to change its length (Figure 6). However, the deformation was restrained by element’s connectivity with other elements in the mesh. This resulted in generation of tensile stresses. Each element, according to its cross section *A*, its local elastic modulus *E*, and the resulting moisture change, was axially loaded with a force:

(11) $$N_{sh}=\varepsilon_{sh}E\;A$$

Once an element broke, it was removed from the mesh. Stresses in that element were released and other elements connected to the broken element were less restrained. During further analysis, new restrain levels and moisture gradients led to stress generation and redistribution among the surviving beams, until the strength of the next element was reached and that element also broke. This is analogue to material shrinkage—once the material cracks, the magnitude of restraints in the material is reduced and the stresses are partially released. Lower restraint levels led to higher deformability of the system and lower stresses.

## 3. Numerical Results and Discussion

In order to verify the proposed modelling approach, crack development and distribution due to restrained shrinkage was examined and compared to damage propagation in experiments. Periodic boundary conditions were applied in two (horizontal) directions (Figure 4). All parameters for moisture transport and fracture properties were taken as given in Table 1 and Table 2.

**Figure 7:**
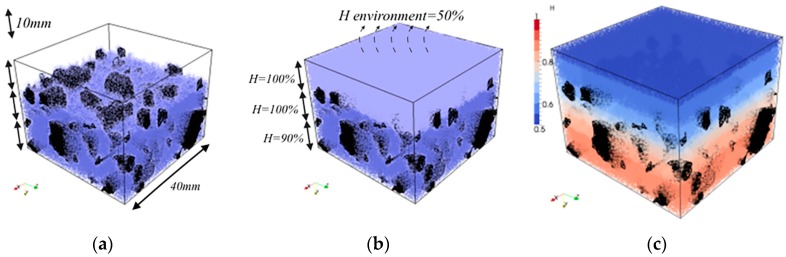
Lattice preparation procedure and moisture simulations (**a**) Imitating sandblasted surface (top 3 mm of aggregates is exposed); (**b**) “Casting” repair mortar with 10 mm thickness; (**c**) Moisture distribution in the repair system at 110 days of drying.

Dimensions of the repair system were 40 × 40 mm^2^ with the total height of 30 mm. Due to high computational demands, specimen dimensions were limited; for the simulation with SHCC, there were in total 639,255 elements (Repair material, RM = 103,752, Mortar Substrate, MS = 144,639, Aggregates = 62,414, ITZ = 43,832, Interface (MS/RM) = 11,731, Interface (Matrix/Fibre) = 183,011, Fibre = 89,876) and 154,045 nodes (48,000 nodes for the repair system without fibres and the rest are additional nodes for fibre elements). Repair material thickness was either 6 or 10 mm. The substrate contained 30% of coarse aggregate particles (by volume), generated and packed using the Anm material model [32]. Top 10 mm of the substrate was fully saturated, while the bottom had an initial relative humidity of 90%. Wet surface of the substrate came either from the wetting of the substrate prior to application of the repair material, or from capillary absorbed water from the repair material. Smooth and sandblasted surface imitated roughness of the concrete substrate (Figure 3a,b). Sandblasted surface was mimicked with 3 mm of aggregates at the surface (Figure 7a). After the substrate surface was “prepared”, the repair material was “cast” on top of it (Figure 7b). Due to drying and substrate absorption, moisture transport in repair system took place (Figure 7c).

**Figure 8:**
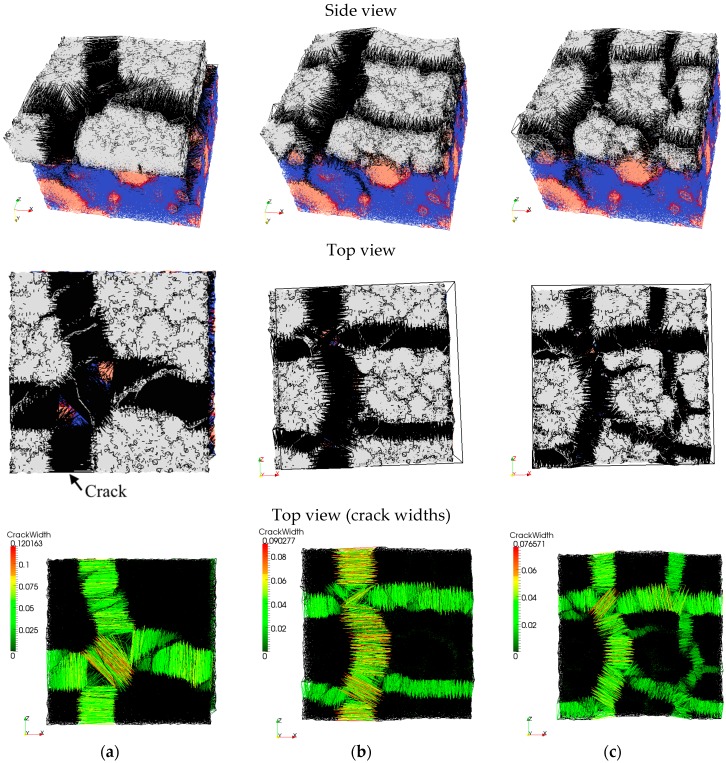
Influence of bond strength and substrate roughness on cracking after 110 days of drying, side and top view: white—repair material, blue—substrate, black—cracks, orange—aggregates, red—ITZ; top view (crack widths): colours as indicated in the legend (**a**) Smooth surface, interface strength 1 MPa; (**b**) Rough surface, interface strength 1 MPa; (**c**) Rough surface, interface strength 3 MPa.

First, non-reinforced repair mortar was used as a repair material. Influence of the interface strength and substrate roughness on the simulated crack pattern at 110 days of drying is shown in Figure 8. Crack width in the lattice model can be calculated for each analysis step. It is defined as an increase in length of the damaged element at a certain analysis step compared to the unloaded state. Debonding in the model (interface crack) is defined as the increase in length of the damaged interface element. In all following images where the damage is shown, deformation is scaled 100×. Maximum crack width and debonding (0.12 mm and 0.056 mm, respectively) are observed when the substrate surface is smooth and the interface strength is 1 MPa (Figure 8a). Cracks in the repair mortar form at close to a 90° angle. A rough surface with the same interface strength enables a similar polygonal pattern with almost straight cracks (Figure 8b). However, due to more restraint and higher friction at the interface, provided by the higher roughness profile, more cracks with smaller crack spacing and smaller crack widths (maximum crack width of 0.090 mm and debonding of 0.044 mm) form. If the bond strength at the interface increases, more cracks are observed (maximum crack width is 0.077 mm and debonding is 0.03 mm) and cracks are not that straight (Figure 8c). Apart from T-junctions (cracks meeting at the angle of 90°), there is also a Y-junction (triplet junction with cracks meeting at the angle of 120°).

Experimentally, Groisman and Kaplan [40] found transition from T-junction to Y-junction cracking when the thickness of drying material was reduced. They reported it to be a consequence of crack nucleation from defects and inhomogeneities in the material when the layer thickness is sufficiently small. Shorlin et al. [41] also reported a few triplet Y-junctions from experiments when sand particles were sprinkled evenly in drying slurry. In presented simulations, sand particles in the repair materials were not explicitly simulated, but inhomogeneity originates from the rough surface of the substrate. The influence of substrate roughness on crack patterns observed at the surface of the repair material increases with higher bond strength and smaller repair material thickness. Therefore, further analyses were performed for different repair mortar thicknesses and bond strengths.

**Figure 9:**
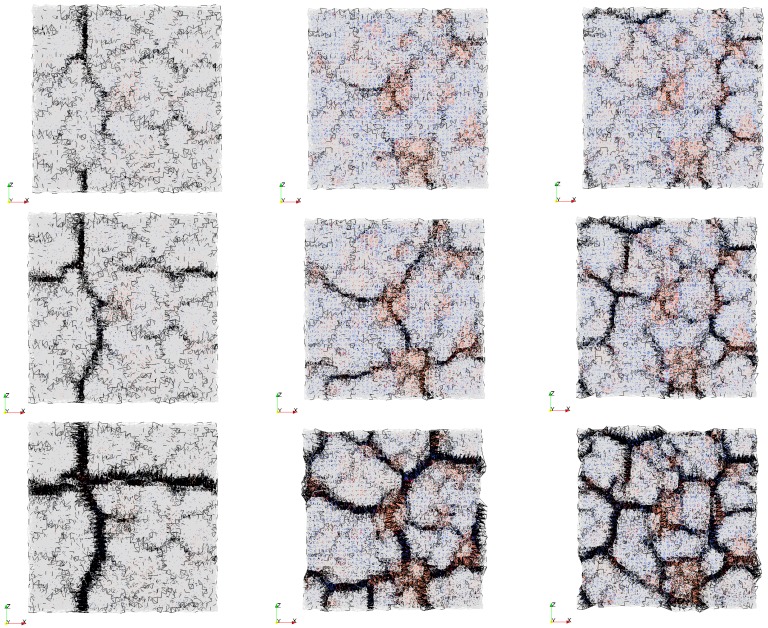

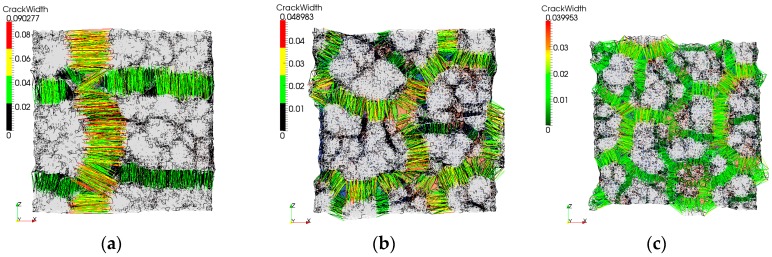
Damage development as a function of time, rough substrate surface, from top to bottom, at 5000, 10,000, 20,000 damaged elements, final crack widths at 110 days of drying (**a**) interface strength 1 MPa and 10 mm thick repair material; (**b**) interface strength 1 MPa and 6 mm repair material thickness and (**c**) interface strength 3 MPa and 6 mm thick repair mortar.

In Figure 9a, crack propagation in the repair mortar with 10 mm thickness is shown. Interface strength between the repair material and the concrete substrate is 1 MPa, which leads to low restraint at the interface. First, a single crack forms. This crack is not affected by successive cracking. However, it determines the boundary conditions for the following crack. The following crack meets the existing crack at close to 90° angle. This is due to the fact that a crack tends to produce the maximum relief of stresses. Therefore, it propagates perpendicular to the direction where the stresses are the highest. Both the second and the third crack initiate from the concave edge of the first crack. This was also observed by Shorlin et al. [41] when they explained why the peak of crack distribution is at slightly more than 90°. In this crack development, cracks form successively one after another and, therefore, subsequent cracks meet the existing cracks at roughly 90°. Cracking sequence in the simulation resembles the experimentally investigated drying shrinkage cracks [41].

In Figure 9b, the repair material thickness was reduced to 6 mm. Interface strength was also set to 1 MPa. It can be observed that immediately a couple of Y-junctions form, mostly above the exposed aggregates in the concrete substrate (orange zones in Figure 9b). These Y-junctions are formed almost simultaneously and later they meet. Similarly, Bohn et al. observed [42] that “starlike cracks with relative angles of 120° occur mainly at material defects in a thin layer and that there is neither a temporal succession of the cracks nor a local geometrical hierarchy”. Maximum crack widths are smaller (in the repair material 0.049 mm, debonding 0.026 mm) compared to those in thicker layer of repair material. In addition, crack patterns from Figure 9a,b confirms that crack spacing is simply proportional to the thickness of the drying layer as was previously stated by Groisman and Kaplan [40]. In their tests, drying coffee-water mixture was tested. A similar trend is also observed for drying damage in other materials [18,41].

Impact of heterogeneity of the substrate roughness on the damage development in the repair material is enhanced by increased bond strength (Figure 9c). Triple Y-junction cracks form, and, due to more restraint at the interface, crack spacing and maximum crack widths are lower. This is in accordance with the experimental results [40,41], where the friction between drying material and substrate was varied (by coating with Vaseline, using Teflon, or drying on a rough sandpaper substrate). In these experiments, bottom friction with the substrate critically influenced the final crack spacing and width. Compared to the simulated repair system, there is an analogy between bond strength and friction with the substrate.

**Figure 10:**
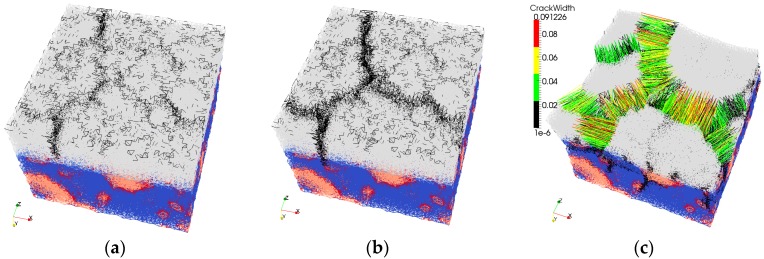
Crack development in the 10 mm thick repair material with smooth surface of substrate and high interface strength (3 MPa) at (**a**) 5000 damaged elements; (**b**) 10000 damaged elements and (**c**) final crack width at the age of 110 days.

In simulations, shift in angle between cracks was also observed for the smooth surface case, when only the bond strength changes. This means that the higher restraint level itself (without heterogeneities in the material or interface) will also contribute to change in the cracking pattern (Figure 10). With smooth surface substrate and high bond strength, stresses in the repair mortar are more uniform. Y-cracks are then preferable considering the “ratio of elastic energy relief to energy created by cracking surface” [40].

As the thickness of the drying layer decreases, more Y-cracks over T-cracks can be observed in an unconfined hardened cement paste sample exposed to drying shrinkage by Bisschop and Wittel [43]. In their study, however, no aggregates were added to the mixture and the tested thicknesses were 3, 4 and 6 mm. All of these thicknesses are at least 30 times larger than the size of a cement particle and, therefore, crack patterns should not be influenced by the ratio between maximum inhomogeneity and thickness of the sample. However, friction at the bottom and therefore higher restraints provided by the reduced thickness are probably sufficient to provide the change of angles in the crack pattern.

**Figure 11:**
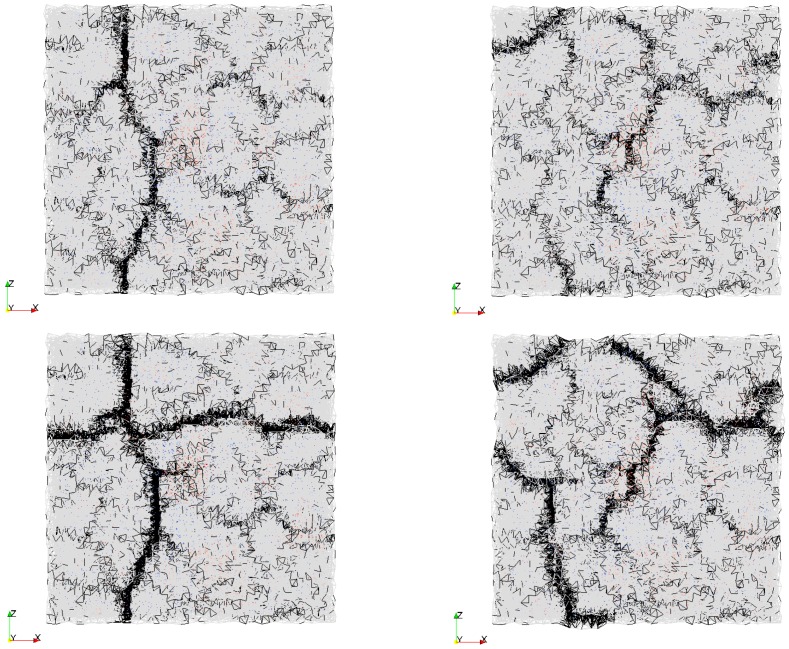

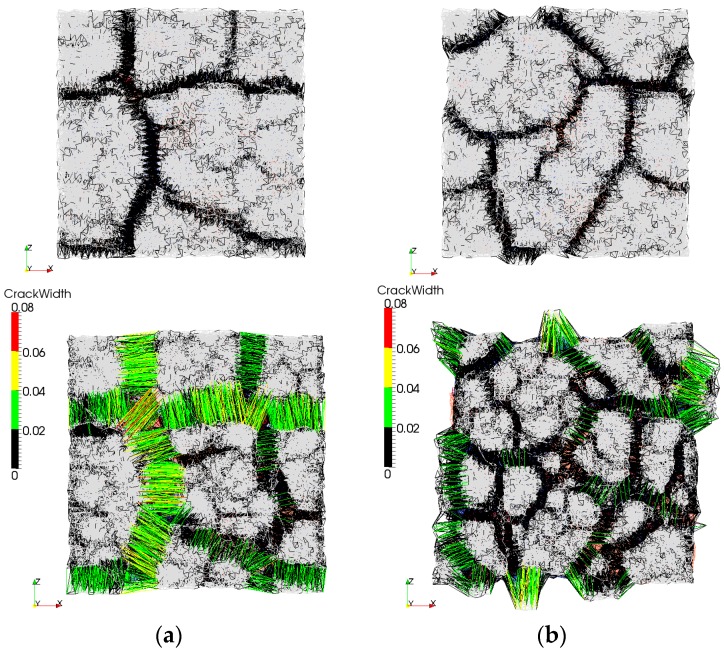
Damage development in repair systems with high interface strength (3 MPa) and a rough substrate surface as a function of time, from top to bottom, at 5000, 10,000 and 20,000 damaged elements, final crack widths at 110 days of drying, repaired with a 10 mm thick (**a**) repair mortar and (**b**) SHCC.

**Figure 12:**
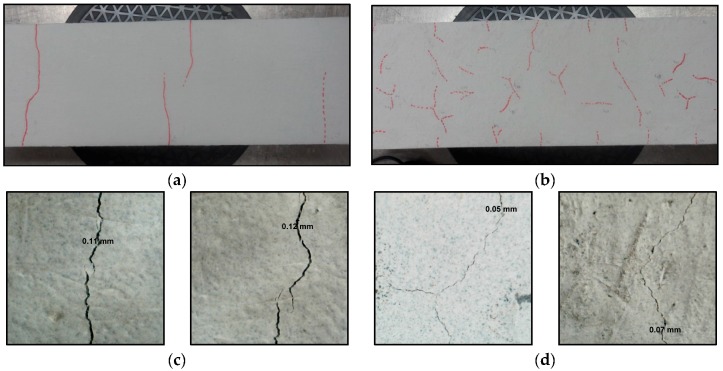
Experiments: Crack patterns in a commercial repair material at (**a**) low and (**c**) high magnification and in strain hardening cementitious composite (SHCC) repair material at (**b**) low and (**d**) high magnification (more results can be found in Chapter 6 in [39]).

With SHCC as a repair material, once the fibres are activated, they arrest the crack (Figure 11b). Simulation results of a regular repair mortar (without fibre reinforcement) and SHCC are given in Figure 11a,b respectively. Both systems have the same transport and mechanical properties. Similar crack patterns are obtained in drying shrinkage experiments, on specimens containing alkali-resistant glass fibres [44]. Fibre alignment and orientation enhances the heterogeneity and the level of restraint in the repair material (similar to sand grains). As a result, more Y-junctions form simultaneously, and later these cracks meet. This is in accordance with findings of Horing et al. [45]: they concluded that, for strong disorder, the system “breaks through the coalescence of initially independent point defects”. On the contrary, for the less disordered material it “breaks through crack propagation”. Apart from introducing higher heterogeneity, fibres also prevent cracks from widening. Therefore, the maximum crack width and crack spacing in SHCC is smaller compared to repair mortar (0.0546 mm compared to 0.077 mm). This is in accordance with the authors’ experiments (Figure 12), in which damage due to drying shrinkage was studied in two repair systems (Chapter 6 in [39]). In Figure 12a,b, crack patterns due to restrained shrinkage in a system with commercial repair mortar and SHCC, respectively, are presented. In Figure 12c,d maximum crack widths in two systems are given.

**Figure 13:**
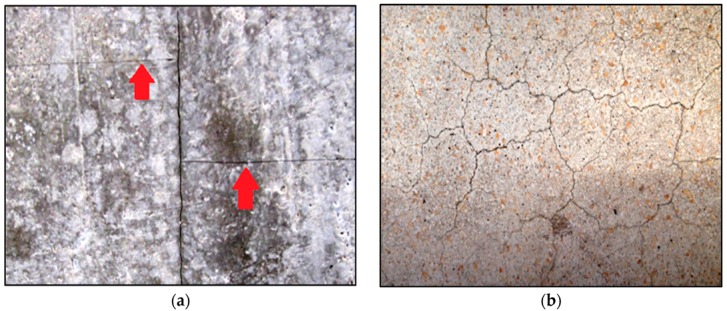
Cracks in concrete (**a**) T-junctions; (**b**) mostly Y-junctions (photos of existing structures).

In essence, aggregate and fibres, or any sort of heterogeneity, introduce local restraint in the material. As the level of heterogeneity increases, cracks become more tortuous and irregular. Y-junctions in the model form simultaneously and subsequently coalesce. On the contrary, T-junctions form successively when a new crack intersects the existing one. Both types of cracks can be found in concrete (Figure 13). Simulated crack patterns closely resemble those found in other homogenous and heterogeneous materials. Furthermore, using the proposed model, additional phenomena can be captured [18,40,41]: crack spacing is proportional to the layer thickness and as the friction with substrate increases (or bond strength) spacing between cracks decreases. Once quantitatively verified with experimental observations, the model can be reliably used to investigate the influence of critical parameters for the repair system performance.

## 4. Conclusions

In the present work, a lattice-based model is proposed to simulate moisture transport and drying shrinkage-induced cracking in the repair systems. The model was compared to experimental observations to evaluate its ability to mimic the processes involved. Different parameters influencing cracking in repair systems caused by drying shrinkage, such as the bond strength, repair material thickness, and type of repair material, have been simulated and evaluated. Based on above findings, the following conclusions can be drawn:

- The influence of repair layer thickness, amount of restraint, and heterogeneity of the repair material on crack pattern (crack spacing, geometry, and angles) due to drying shrinkage can be captured with the presented modelling approach.
- If there is no continuous delamination, with the same bond strength and drying conditions, more cracks (with lower crack spacing) are observed in thinner overlays. The number of cracks increases with an increase in bond strength or substrate roughness. This is in accordance with experimental observations in drying shrinkage tests of different brittle and quasi-brittle materials.
- Y-junctions in the lattice model form simultaneously as a consequence of higher heterogeneities (defects or inclusions, surface roughness of the substrate) and higher restraint by the substrate (smaller thickness, high bond strength). On the contrary, T-junctions are formed successively, when one crack intersect an existing crack (bigger thickness, lower bond strength).
- With adequate bonding, SHCC performs better in simulated drying shrinkage tests. It exhibits small crack widths which is beneficial for durability properties of repair systems. Repair material should have strain-hardening behaviour in order to enable multiple crack formation and to prevent wide opening of the crack.

The failure mode of the repair system due to drying shrinkage is very sensitive to substrate surface roughness, thickness of the overlay and moisture gradients due to drying. Simulation results show that, in general, performance of the repair system due to drying shrinkage can be well imitated. In the models, however, a number of assumptions are made regarding the input parameters. Diffusivity parameters and hygral coefficients used as inputs are, in reality, different for every repair material and concrete substrate. Therefore, they should be measured for every mixture, as they influence the state of stresses in the repair system. Due to difficulties in experimental quantification of the diffusivity and fracture properties of interfaces, values used in the model are only reasonable assumptions.

Edge boundary conditions were not included in the model. However, it was considered that, after cracking, boundary conditions around this crack represent edge effect in a repair system.

Time dependency was not taken into account in simulations. In reality, material properties (diffusion coefficient, modulus of elasticity, strength) are time dependent. In this research only shrinkage strains were considered as a function of time, and they were fitted such that they correspond to experimentally measured values. No change in diffusion coefficient, modulus of elasticity, or strength was considered. In future research, time (hydration) dependency, should also be included.

Due to stress relaxation in the repair material, the magnitude of stresses due to internal and external restraints will be lower and, therefore, crack initiation will be postponed. Because, in case of a repair system, relaxation has a beneficial influence, neglecting its influence is a conservative approach when determining the performance of repair systems. For a more accurate prediction and quantification of the repair system performance, both the relaxation of the repair material and the creep of the substrate need to be considered. As long as the same fracture behaviour (crack widths, spacing, debonding) of repair systems in experiments and simulations is obtained as a function of time, maybe it can be (roughly) considered that these relaxation and creep are implicitly taken into account.

## Figures and Tables

**Table 1:** Diffusivity parameters used in the lattice moisture transport model to obtain diffusion coefficient, *D* (mm^2^/day) (Equation (2)). Parameters are adjusted such that the moisture profiles in the repair system at 1 day, 10 days and 110 days are the same as those obtained by Wittmann and Martinola [12].

Diffusivity Parameters	Repair Mortar	Interface	Mortar Substrate	ITZ	Aggregates
*β*	0.022	0.022	0.022	0.066	0.00022
*γ*	7.5	7	4	4	0

**Table 2:** Input values for lattice fracture model [27], *E* stands for the modulus of elasticity, *f_t_* stands for the tensile strength and *f_c_* for the compressive strength of lattice elements.

Element		*E* (GPa)	*f_t_* (MPa)	*f_c_* (MPa)
Matrix (repair mortar-RM)		20	3.5	35
Fibre		40	7380	(7380)
Interface (Matrix/Fibre)		20	90	900
Mortar substrate		25	4	40
Aggregate		70	8	80
ITZ		15	2.5	25
Matrix (Repair mortar-RM)		20	3.5	35
Interface (Matrix/fibre)		20	90	900
Interface (MS/RM)	Weak	15	1	10
	Strong	15	3	30
